# 180. Duration of Therapy and Clinical Outcomes in Adult Oncology Patients with Uncomplicated Coagulase Negative Staphylococcal Bacteremia

**DOI:** 10.1093/ofid/ofab466.382

**Published:** 2021-12-04

**Authors:** Amanda Fairbanks, Jessica Li, Ania Sweet, Frank Tverdek, Catherine Liu

**Affiliations:** 1 University of Washington, School of Pharmacy, SEATTLE, Washington; 2 University of Washington, Department of Pharmacy, Seattle, Washington; 3 Seattle Cancer Care Alliance, University of Washington, Fred Hutch Cancer Research Center, Sammamish, Washington; 4 Fred Hutchinson Cancer Research Center, Seattle, Washington; 5 Fred Hutchinson Cancer Research Center; University of Washington, Seattle, Washington

## Abstract

**Background:**

Although they are often considered contaminants, coagulase negative staphylococci (CoNS) can be pathogens especially in immunocompromised patients. National and local guidelines recommend treatment durations of 7 to 14 days, depending on specific clinical scenarios. The objective was to characterize the duration of treatment for CoNS bacteremia and clinical outcomes at our cancer center.

**Methods:**

We conducted a retrospective chart review of adult patients ≥18 years old with ≥1 blood culture with growth of CoNS between 1/1/17 and 12/31/19 at our cancer center. Patients with complicated CoNS bacteremia and polymicrobial infections were excluded.

**Results:**

Among 128 patients identified during the study period, 98 met inclusion criteria (Figure 1). Most patients (N= 92; 94%) had a hematologic malignancy as the underlying oncologic diagnosis, and 68 (69%) were hematopoietic stem cell transplant recipients. The median total antibiotic duration was 13 days, and median duration from the date of 1^st^ negative blood culture was 12 days; 29 (30%) patients were treated for a total duration of >14 days (Figure 2). The catheter was retained in 67 (68%) and exchanged in 4 (4%) of the cases. Three (3%) patients had recurrence of bacteremia within 30 days of treatment completion, and 8 (8%) patients were transferred to the ICU within 7 days of the index blood culture. The 30-day crude mortality rate was 10%. The most commonly used antibiotic for treatment was vancomycin (N= 95; 97%), and 32 (34%) patients on vancomycin had an increase in serum creatinine of ≥ 50% from baseline. Five (5%) patients discontinued vancomycin due to nephrotoxicity, and 4 (4%) patients required hemodialysis.

Figure 1. Results Overview

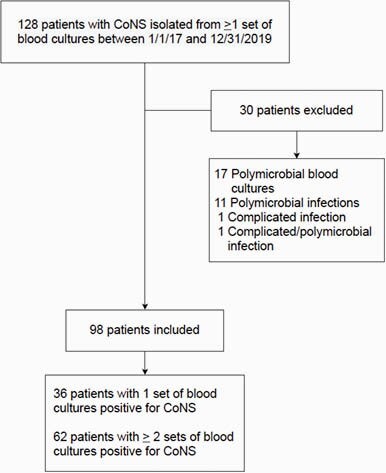

Among 128 patients identified during the study period, 98 met inclusion criteria. Of those included, 36 had one set of blood cultures that were positive for CoNS and 62 patients had at least two sets of blood cultures that were positive for CoNS.

Figure 2. Duration of Antibiotic Treatment

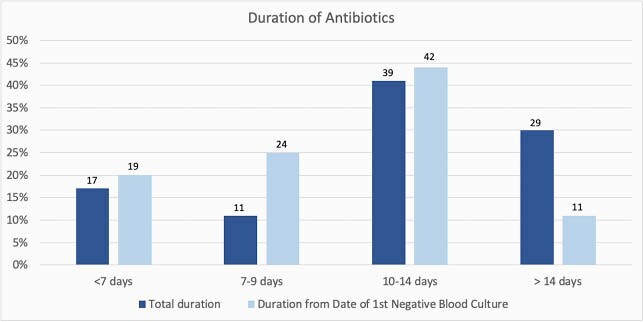

We evaluated duration of treatment based on total antibiotic duration and duration from date of 1st negative blood culture. The number of patients is noted above each bar.

**Conclusion:**

Although the majority of patients were treated for ≤14 days for uncomplicated CoNS bacteremia, nearly 1/3 of patients were treated for > 14 days. Recurrent bacteremia was uncommon despite catheter retention in most patients. Relatively high rates of vancomycin associated nephrotoxicity highlight opportunities for antimicrobial stewardship to limit duration of vancomycin therapy among cancer patients with uncomplicated CoNS bacteremia.

**Disclosures:**

**All Authors**: No reported disclosures

